# Multiple myeloma cells alter the senescence phenotype of bone marrow mesenchymal stromal cells under participation of the DLK1-DIO3 genomic region

**DOI:** 10.1186/s12885-015-1078-3

**Published:** 2015-02-18

**Authors:** Rimma Berenstein, Olga Blau, Axel Nogai, Marlies Waechter, Ekaterina Slonova, Martin Schmidt-Hieber, Annegret Kunitz, Antonio Pezzutto, Bernd Doerken, Igor Wolfgang Blau

**Affiliations:** 1 Department of Hematology, Oncology and Tumourimmunology, Charité Universitätsmedizin Berlin, Hindenburgdamm 30, 12200 Berlin, Germany; 2 Department of Hematology, Oncology and Tumourimmunology, Helios Clinic Berlin-Buch, Berlin, Germany

**Keywords:** Multiple myeloma, Bone marrow stromal cells, Senescence, Cell cycle, DLK1-DIO3, miR-485-5p

## Abstract

**Background:**

Alterations and senescence in bone marrow mesenchymal stromal cells of multiple myeloma patients (MM-BMMSCs) have become an important research focus. However the role of senescence in the pathophysiology of MM is not clear.

**Methods:**

Correlation between senescence, cell cycle and microRNA expression of MM-BMMSCs (n = 89) was analyzed. Gene expression analysis, copy number analysis and methylation specific PCR were performed by Real-Time PCR. Furthermore, cyclin E1, cyclin D1, p16 and p21 genes were analyzed at the protein level using ELISA. Cell cycle and senescence were analyzed by FACS. MiRNA transfection was performed with miR-485-5p inhibitor and mimic followed by downstream analysis of senescence and cell cycle characteristics of MM-BMMSCs. Results were analyzed by Mann–Whitney U test, Wilcoxon signed-rank test and paired t-test depending on the experimental set up.

**Results:**

MM-BMMSCs displayed increased senescence associated β-galactosidase activity (SA-βGalA), cell cycle arrest in S phase and overexpression of microRNAs. The overexpressed microRNAs miR-485-5p and miR-519d are located on DLK1-DIO3 and C19MC, respectively. Analyses revealed copy number accumulation and hypomethylation of both clusters. KMS12-PE myeloma cells decreased SA-βGalA and influenced cell cycle characteristics of MM-BMMSCs. MiR-485-5p was significantly decreased in co-cultured MM-BMMSCs in connection with an increased methylation of DLK1-DIO3. Modification of miR-485-5p levels using microRNA mimic or inhibitor altered senescence and cell cycle characteristics of MM-BMMSCs.

**Conclusions:**

Here, we show for the first time that MM-BMMSCs have aberrant methylation and copy number of the DLK1-DIO3 and C19MC genomic region. Furthermore, this is the first study pointing that multiple myeloma cells in vitro reduce both the senescence phenotype of MM-BMMSCs and the expression of miR-223 and miR-485-5p. Thus, it is questionable whether senescence of MM-BMMSCs plays a pathological role in active multiple myeloma or is more important when cell interaction with myeloma cells is inhibited. Furthermore, we found that MiR-485-5p, which is located on the DLK1-DIO3 cluster, seems to participate in the regulation of senescence status and cell cycle characteristics of MM-BMMSCs. Thus, further exploration of the microRNAs of DLK1-DIO3 could provide further insights into the origin of the senescence state and its reversal in MM-BMMSCs.

**Electronic supplementary material:**

The online version of this article (doi:10.1186/s12885-015-1078-3) contains supplementary material, which is available to authorized users.

## Background

Multiple Myeloma (MM) is a B-cell malignancy characterized by the accumulation of malignant plasma cells (PC) within the bone marrow (BM) and the strong interaction between several cellular compartments [1].

BMMSCs support MM cell growth through different direct and indirect factors leading to increased tumor support and possible generation of drug resistance [2-10].

Thus, the surrounding tumor microenvironment has become a focal point of MM research. Several studies have suggested the genesis of constitutive abnormalities in MM-BMMSCs through interactions with MM cells [11-14]. For instance development of a senescence-like state in BMMSCs and thereby a modulated secretory profile, worsened osteogenic differentiation potential and inhibition of the T-cell proliferation were reported [13,15]. Senescence is a cellular state associated with the loss of proliferative capacity and changes in the secretion of pro-inflammatory cytokines and growth factors [16]. Senescent BMMSCs display an increased senescence-associated β-galactosidase activity (SA-βGalA) and irregular cell morphology. Usually the cell cycle of senescent cells is arrested at the G_1_/S-transition point in combination with the overexpression of different cell cycle inhibitors as p21 and p16. In spite of the aberrant growth characteristics senescent cells remain metabolically active and therefore the secretion of pro-inflammatory mediators could promote tumorigenesis in neighboring premalignant cells [17-19]. Although some reports describe constitutive changes in MM-BMMSCs, the molecular mechanisms and pathways that induce senescence-associated abnormalities are largely unknown. Furthermore it is not clear whether alterations of MM-BMMSCs are important for the interaction between stromal cells and MM cells or are more an attendant phenomenon.

Two imprinted clusters in the human genome might contribute to the generation of senescence and induction of cellular changes in MM-BMMSCs [20-23]. The DLK1-DIO3 imprinted domain is located on chromosome 14q32.2 and expresses 53 microRNAs, whereas the imprinted cluster C19MC is located on chromosome 19q13 and codes for 59 microRNAs [24-26]. Allelic expression of DLK1-DIO3 is controlled through methylation of a regulatory region (IG-DMR) located about 15 kb upstream of the cluster and the expression of C19MC correlates with the epigenetic modulation of a CpG-rich region located about 17.6 kb upstream [26,27].

Up to now no data on the role of the senescent phenotype of MM-BMMSCs for the progression of MM are available. Previously, we have shown that MM-BMMSCs exhibit overexpression of distinct microRNAs and an increased senescence phenotype as compared to healthy donor BMMSCs [28]. To further address this point we analyzed in this study the correlation between senescence status, cell cycle characteristics and microRNA expression of MM-BMMSCs. We chose microRNAs, which were previously reported to be deregulated in MM cells and play a potential role in inflammation-induced cellular senescence [21,29-32]. We further addressed the question of whether interaction of MM-BMMSCs with MM cells could modulate the cell cycle and senescence-like state of MM-BMMSCs by altering the expression of microRNA molecules. In this context we wanted to analyze whether the premature senescence status of MM-BMMSCs could be a specific effect induced by MM cells for increased tumor support. MiR-485-5p, which is encoded by the DLK1-DIO3 cluster, was found to be a potential modulator of the cell cycle and senescence status of MM-BMMSCs. Based on this observation we finally investigated the effect of miR-485-5p mimics or inhibitor on the cell cycle and SA-βGalA of MM-BMMSCs.

## Methods

### Patient and donor characteristics

BM samples from 89 patients with multiple myeloma [n = 54 with MM at the time of diagnosis and n = 35 at relapse] were included in the study. All patients had a treatment indication. Written informed consent was obtained from all patients and healthy donors in accordance with the Declaration of Helsinki and the ethical guidelines of the Charite University School of Medicine, which approved this study (Votum No.: EA4/131/13). Twelve bone marrow aspirates were received from healthy donors (see Additional file 1: Table S1: Patients and Donor Characteristics).

### Isolation and cultivation of BMMSCs

BMMSCs from patients with MM (MM-BMMSCs) or donors (HD-BMMSCs) were isolated by the classical adhesion method and cultivated as previously described [33-35]. Non-hematopoietic cell characteristics were identified by flow cytometry by absence of CD45 and CD34 and presence of CD105 and CD90 expression with antibodies from Miltenyi Biotec. Data was acquired and analyzed with a FACS Calibur Flow Cytometer (BD Biosciences) and Flowing Software (Cell Imaging Core). Purity of isolated BMMSCs ranged from 94% to 99.5% (see Additional file 2: Figure S1: Purity of isolated BMMSCs).

### Co-Cultures and transwell cultures of MM-BMMSCs

For co-cultures 5×10^4^ MM-BMMSCs were seeded in a 6-well plate and incubated for 4 h. Then 5×10^4^ KMS12-PE myeloma cells were added followed by incubation for 72 h. After incubation KMS12-PE cells were removed by rigorous pipetting and detachment was confirmed by microscopy. In addition the absence of CD138^+^ cells was checked with FACS during cell cycle analysis. MM-BMMSCs were washed twice with PBS and applied for downstream analysis. Co-cultured KMS12-PE myeloma cells were pelleted and re-suspended in TRIzol for downstream analysis.

For transwell cultures (0.4 μM pore size, Corning) 2×10^4^ MM-BMMSCs were seeded in the lower chamber of a 12-well plate and incubated for 4 h. Then 2×10^4^ KMS12-PE myeloma cells were added to the upper chamber. Incubation was performed for 72 h.

Cultures without KMS12-PE cells served as negative control for transwell cultures and co-cultures.

### Detection of SA-βGalA

β-galactosidase activity was measured as previously reported [36]. Data was acquired as described above and analyzed using the median fluorescence intensity (MFI). Co-cultures of HD-BMMSCs (n = 3) and HS-5 stromal cells (n = 3, CRL-11882) were used as controls. In addition β-galactosidase activity was analyzed using the “Senescence cells Histochemical Staining Kit” (Sigma Aldrich) as recommended by the manufacturer.

### Cell cycle analysis

Cell analysis was performed using the “Cell cycle Assay Kit” (Abcam) as recommended by the manufacturer. Data was acquired using a logarithmic scale.

### Quantitative Real-Time PCR (qPCR)

Total RNA was extracted using TRIzol as described previously [37]. RNA was treated with DNase (Ambion) and poly(A)-polymerase (NEB) according to the manufacturer’s instructions. 800 ng of RNA was used for cDNA synthesis with a Transcriptor First Strand cDNA Synthesis Kit (Roche) and 2 μl of poly(T)VN adaptor primer (10 pmol) in a 20 μl reaction.

qPCR was performed with the FastStart Universal SybrGreen Master Mix (Roche). MicroRNA detection was performed as described [38]. GAP-DH and 5.8S rRNA were chosen as housekeeping genes. All primers and corresponding accession numbers are listed in Additional file 3: Supplemental Methods; Table S2. QPCR was carried out with the RotorGene 6000 Real-Time PCR cycler using 1:5 diluted cDNA (8 ng) as template. Cycling conditions are described in Additional file 2: Supplemental Methods; Table S3. qPCR efficiencies were determined using linear regression analysis [39,40] using LinRegPCR software and relative quantifications were estimated with the Pfaffl-method [41]. Received data was analyzed with the Rotor Gene 6000 software.

### Quantitative methylation-specific PCR (qMSP)

DNA isolation was performed using Puregene reagents (Qiagen) according to the manufacturer’s instructions. 300 ng of genomic DNA was subjected to bisulfite treatment with the EpiTect Fast Bisulfite Conversion Kit (Qiagen) as recommended in the manual. Primers are described in Additional file 2: Supplemental Methods; Table S2. Reactions were performed with 30 ng treated DNA using SYBR Green Master Mix (Roche). Thermal conditions were as described above. Quantification was carried out using a standard curve generated using a dilution series of fully methylated with unmethylated DNA (Applied Biosystems). Each sample was analyzed in duplicates and Ct-values above 32 were excluded.

### Copy number (CN) variation assay

Three genomic regions located along each of the clusters were chosen for CN estimations of DLK1-DIO3 and C19MC. Assay numbers (qBiomarker Copy Number Assays, Qiagen) are listed in Additional file 2: Supplemental Methods; Table S4. Genomic DNA of the stromal cell line HS-5 (CRL-11882) was applied as a calibrator. Cycling was carried out as recommended by the manufacturer. Relative quantification was achieved by the ΔΔC_t_ method as described in the qBiomarker manual.

### microRNA mimic and inhibitor experiments

MM-BMMSCs were seeded in a 6-well plate with 1–2.5*10^5^ cells/well. After incubation for 3 h cells were transfected with 10 mM of miR-485-5p mimic, 100 mM of miR-485-5p inhibitor or the appropriate negative control. The mimic, inhibitor and corresponding negative controls were all obtained from Qiagen AG. Transfections were performed with the HiPerfect Transfection Reagent (Qiagen) as recommended by the manufacturer. In addition, a transfection control containing only the transfection reagent was carried out. Transfected MM-BMMSCs were incubated for 48 h before use for downstream analysis.

### Indirect enzyme-linked immunosorbent assay (ELISA)

Complete cell lysates of BMMSCs were prepared using RIPA buffer (Pierce). The protein amount was detected with a BCA Protein Assay Kit (Pierce). 5 μg of total protein were used for ELISAs. 96-well plates were coated with the samples overnight at 4°C using BupH coating buffer (Pierce). The coated wells were blocked for 1 h at room temperature (5% nonfat dry milk in PBS). Primary antibodies were diluted in blocking buffer and incubation was performed overnight at 4°C (cyclin D1 and p21 1:500 (Cell Signaling); cyclin E (sc-481) 1:100 (Santa Cruz); CDKN2A 1:200 (Thermo Scientific)). Between the incubation steps the wells were washed three times with PBS containing 0.1% Tween. The secondary antibody (anti-rabbit IgG-HRP (Cell Signaling)) was diluted 1:1000 and incubation was performed for 2 h at room temperature. Detection was performed with TMB substrate (Pierce) and absorption was measured at 450 nm. All measurements were performed with three technical replicates. A dilution series of complete cell lysates of the HS-5 cell line was used for standard curve generation enabling relative quantification of protein levels. Pure RIPA buffer served as negative control.

### Statistical analysis

All experiments were statistically analyzed using GraphPad Prism 6 software (LA Jolla, CA, USA). The data shown represents the mean ± standard error of the mean (SEM). Comparisons of HD-BMMSCs with MM-BMMSCs were performed using the Mann–Whitney U test. The Wilcoxon signed-rank test was used for the analysis of co-cultures and transwell cultures. The unpaired/paired t-test was used for statistical analysis of protein level results (ELISA analysis). Transfection experiments were analyzed using the paired t-test. Results were considered statistically significant when p ≤ 0.05.

## Results

BMMSCs from MM patients at the time of diagnosis are referred to as ND-MM-BMMSCs and BMMSCs from relapsed MM patients are referred to as R-MM-BMMSCs. For both analysis groups the abbreviation MM-BMMSCs is used. BMMSCs from healthy donors are referred to as HD-BMMSCs. Because ND-MM-BMMSCs and R-MM-BMMSCs showed a similar mRNA expression, no separate analysis of ND-MM-BMMSCs and R-MM-BMMSCs was performed for protein tests.

Given that the observed changes in ND-MM-BMMSCs and R-MM-BMMSCs upon co-cultivation with KMS12-PE myeloma cells were similar the data of co-cultures, transwell cultures and microRNA transfection experiments of both groups were combined to one analysis group (MM-BMMSCs).

### MM-BMMSCs exhibit a higher senescence state than HD-BMMSCs

**Figure 1:**
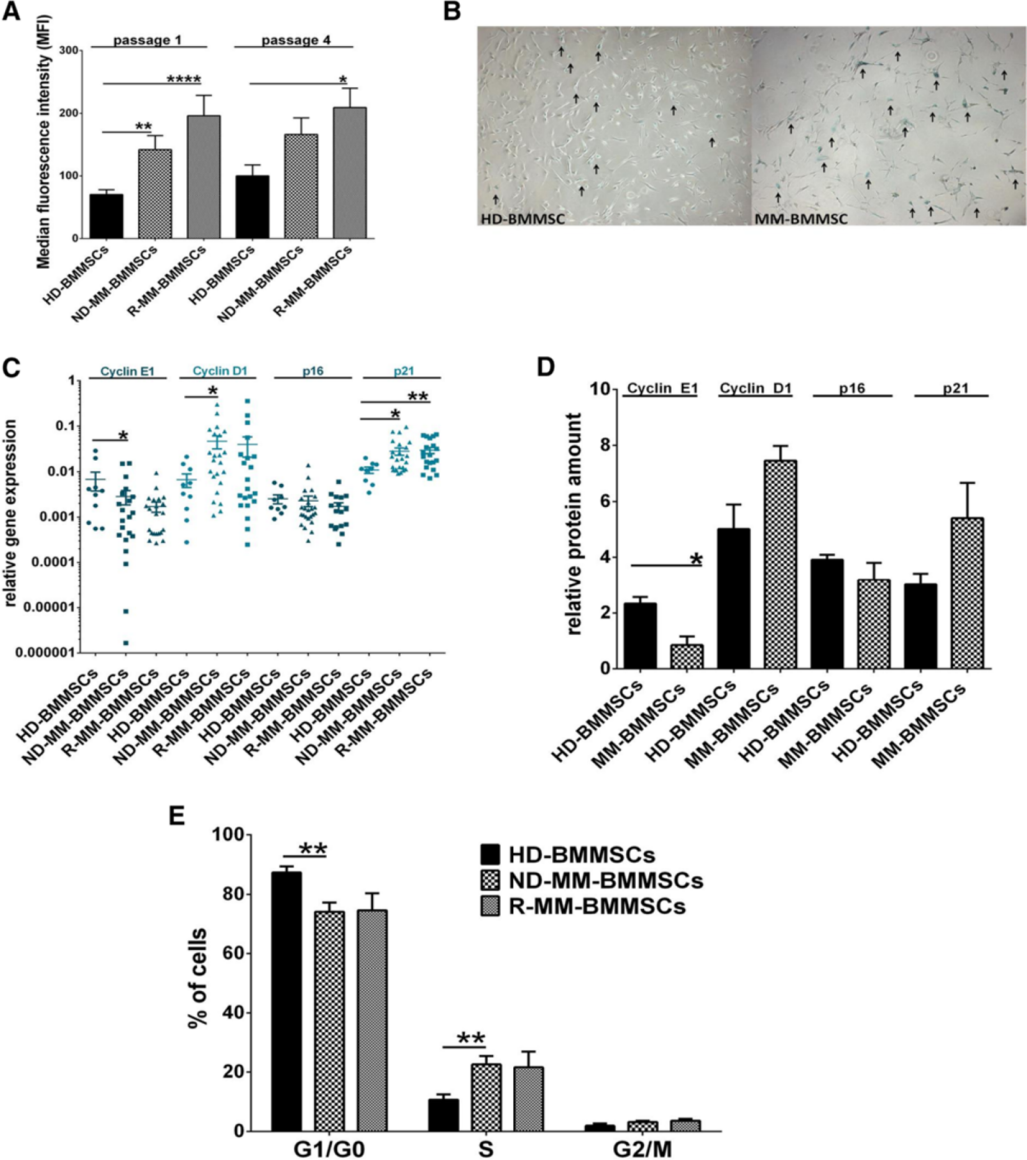
**MM-BMMSCs exhibit a higher senescence state than HD-BMMSCs.** Asterisks indicate p-values with * <0.05, ** < 0.01, *** < 0.001 and **** < 0.0001. All data were analyzed using Mann–Whitney U test and unpaired t-test (ELISA analysis). **(A)** Flow cytometric analysis of SA-βGalA. ND-MM-BMMSCs (n = 12) and R-MM-BMMSCs (n = 9) displayed higher activity of SA-βGal in passage 1 and 4 of cell cultures compared to HD-BMMSCs (n = 8). **(B)** Histological analysis of SA-βGalA. MM-BMMSC (59 years) showed aproximately 17 cells with high SA-βGalA compared to HD-BMMSC (67 years) with only 10 cells. Positive cells are indicated by arrows. **(C)** QPCR analysis displayed decreased cyclin E1, increased cyclin D1 and p21 expression in ND-MM-BMMSCs (n = 24) and R-MM-BMMSCs (n = 21) compared to HD-BMMSCs (n = 10). **(D)** Measurement of protein level in HD-BMMSCs (n = 2) and MM-BMMSCs (n = 3). Cyclin E1 was significantly decreased in MM-BMMSCs compared to HD-BMMSCs whereas cyclin D1 and p21 were increased. The protein amount of p16 was slightly reduced in MM-BMMSCs compared to HD-BMMSCs. **(E)** Cell cycle analysis showed a higher amount of cell in S phase in ND-MM-BMMSCs (n = 11) and R-MM-BMMSCs (n = 5) compared to HD-BMMSCs (n = 6). Percentage of cells in G_1_/G_0_ phase was lower compared to HD-BMMSCs.

In contrast to HD-BMMSCs, MM-BMMSCs displayed a 2- to 3-fold (*p <* 0.005) higher SA-βGalA in passage 1 and 4 of the cell cultures (Figure 1A). R-MM-BMMSCs had a 1.4-fold increased SA-βGalA as compared to ND-MM-BMMSCs. These results could be confirmed by a histological β-galactosidase staining of HD-BMMSCs and MM-BMMSCs in passage 4 (Figure 1B). Furthermore, qPCR analyses showed a 2.5- to 4-fold lower expression of cyclin E1 and a 5- to 6-fold overexpression of cyclin D1 in ND-MM-BMMSCs and R-MM-BMMSCs compared to HD-BMMSCs (*p <* 0.05; Figure 1C). In addition, the cell cycle inhibitor p21 was 2.5-fold upregulated in MM-BMMSCs compared to HD-BMMSCs (*p <* 0.05) against what no changes in the mRNA level of p16 were detected. Similar differences between HD-BMMSCs and MM-BMMSCs were detected at the protein level (Figure 1D). Cyclin E1 was 2.8-fold decreased in MM-BMMSCs compared to HD-BMMSCs (*p* = 0.0416). In contrast, cyclin D1 and p21 protein levels were 1.5-fold to 1.8-fold increased. Protein measurement showed also a slightly reduced level of p16 in MM-BMMSCs but this change was below 1.5-fold. These results correlated with a higher amount of cells in S phase and a reduced amount of cells in G_1_/G_0_ phase compared to HD-BMMSCs (*p <* 0.008; Figure 1E).

### MicroRNAs in MM-BMMSCs are aberrantly expressed

**Figure 2:**
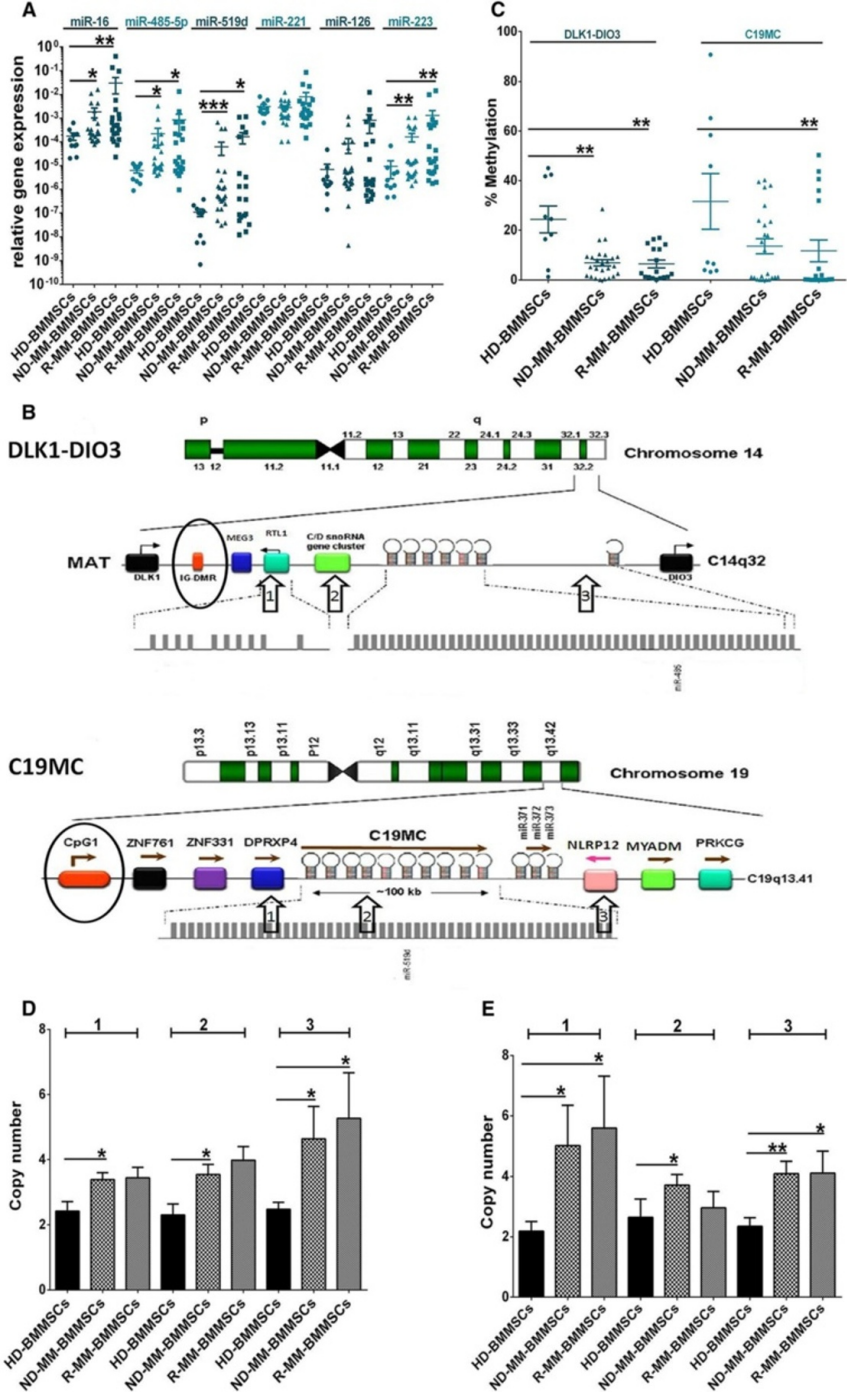
**Overexpressed microRNAs in MM-BMMSCs are associated with hypomethylation and CN accumulation of DLK1-DIO3 and C19MC.** Asterisks indicate p-values with * <0.05, ** < 0.01, *** < 0.001 and **** < 0.0001. All data were analyzed using Mann–Whitney U test. **(A)** ND-MM-BMMSCs (n = 24) and R-MM-BMMSCs (n = 21) showed high overexpression of miR-16, miR-485-5p, miR-519d and miR-223 compared to HD-BMMSCs (n = 10). **(B)** Schematic presentation of the genomic organization of DLK1-DIO3 on chromosome 14 (C14q32) and C19MC on chromosome 19 (C19q13.41). Circles indicate the regulatory region of the respective cluster and arrows point to positions of CN measurement. Modified from Morales-Prieto et al. [27] **(C)** The regulatory regions of DLK1-DIO3 and C19MC were hypomethylated in ND-MM-BMMSCs (n = 25) and R-MM-BMMSCs (n = 18) compared to HD-BMMSCs (n = 9). **(D)** CN analysis of C19MC displayed CN accumulation in all three regions in ND-MM-BMMSCs (n = 23) and R-MM-BMMSCs (n = 15) compared to HD-BMMSCs (n = 8). **(E)** CN analysis of DLK1-DIO3 displayed CN accumulation in all three measured positions in MM-BMMSCs compared to HD-BMMSCs (sample number as indicated in **(D)**). No accumulation was measured for R-MM-BMMSCs in position 2.

To investigate whether senescence of MM-BMMSCs is correlated with changes in the microRNA expression, the level of different microRNAs was analyzed using qPCR. We chose six microRNAs, which were previously reported to be deregulated in MM cells and to play a possible role in the generation of senescence or cell cycle arrest (miR-16, miR-485-5p, miR-519d, miR-221, miR-126, miR-223). Analysis revealed an overexpression of miR-16, miR-223, miR-485-5p and miR-519d (all with *p <* 0.025) in MM-BMMSCs compared to HD-BMMSCs. No expression differences were detected for miR-221 and miR-126 (Figure 2A).

### MM-BMMSCs show hypomethylation and copy number accumulation of the DLK1-DIO3 and C19MC genomic clusters

qPCR analysis revealed the overexpression of miR-485-5p and miR-519d in MM-BMMSCs. These microRNAs are located on two imprinted clusters on chromosomes 14 (DLK1-DIO3) and 19 (C19MC), respectively, and are reported to play a role in senescence generation [21,31,32]. Given that expression of both clusters is controlled by methylation of their regulatory regions, we analyzed their methylation status using qMSP (Figure 2B).

Hypomethylation of both clusters in MM-BMMSCs compared to HD-BMMSCs was observed (Figure 2C). For DLK1-DIO3, MM-BMMSCs exhibited an approximate 5-fold lower methylation level of the IG-DMR. ND-BMMSCs methylation levels were not statistically different for C19MC (*p =* 0.0537). However, they exhibited the same characteristics as for R-MM-BMMSCs (*p =* 0.0062) with a 2.5-fold lower methylation level compared to HD-BMMSCs.

Another reason for the overexpression of miR-485-5p and miR-519d could be CN variations of C19MC and DLK1-DIO3. CN variation analysis was carried out at three regions along each cluster (Figure 2B).

MM-BMMSCs displayed high levels of amplification of the backward region of C19MC (Figure 2D). Error bars indicate the detection of individual MM-BMMSCs with a CN >7. The front and middle regions of C19MC exhibited 3 to 4 copies in MM-BMMSCs whereas in HD-BMMSCs detected CNs were below 2.7 (*p <* 0.05). DLK1-DIO3 was also commonly amplified, especially the front and the back regions (Figure 2E). Copy number alterations for R-MM-BMMSCs in the middle region of DLK1-DIO3 were not detectable (*p = 0*.6466), though ND-MM-BMMSCs exhibited significant amplification (*p =* 0.0065).

### KMS12-PE myeloma cells reduce SA-βGalA and modify cell cycle characteristics of MM-BMMSCs

**Figure 3:**
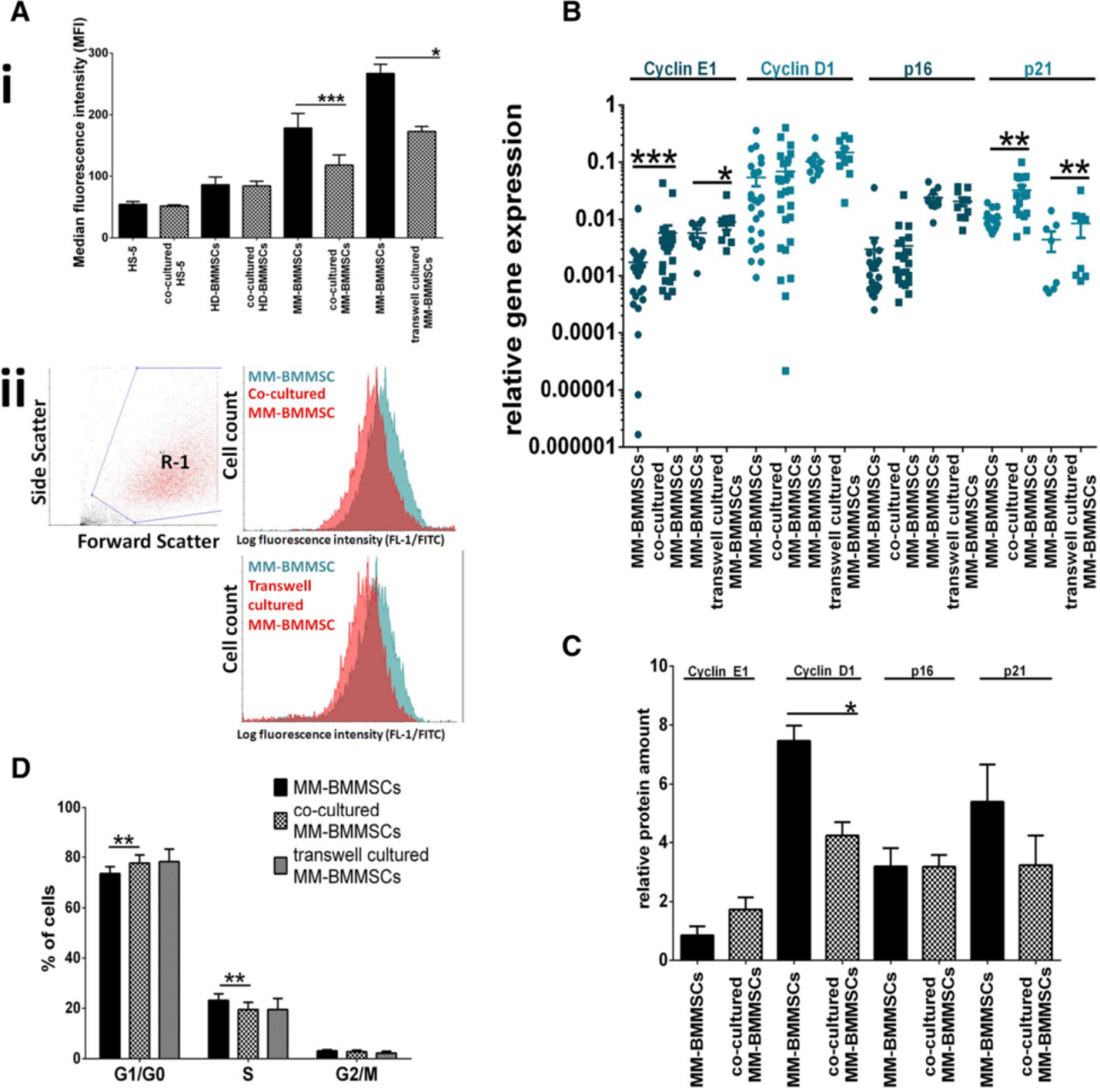
**KMS12-PE myeloma cells reduce SA-βGalA and modify cell cycle characteristics of MM-BMMSCs.** Asterisks indicate indicate p-values with * <0.05, ** < 0.01, *** < 0.001 and **** < 0.0001. All data were analyzed using Wilcoxon signed-rank test and paired t-test (ELISA analysis). **(A)** KMS12-PE myeloma cells reduce SA-βGalA in MM-BMMSCs upon co-cultivation (n = 20) and cultivation in transwells (n = 6). (i) The MFI in MM-BMMSCs was significantly reduced in both culture systems. No changes were observed for co-cultured HD-BMMSCs (n = 3) and HS-5 cells (n = 3) indicating specificity of the measured effect for MM-BMMSCs. (ii) Representative histogram of a co-cultured MM-BMMSC and transwell-cultured MM-BMMSC population compared to the same MM-BMMSC population cultured alone. **(B)** Cell interaction with KMS12-PE myeloma cells induced increased cyclin E1 and p21 expression in MM-BMMSCs compared to MM-BMMSCs cultured alone (co-culture n = 25, transwell culture n = 10). **(C)** Protein expression analysis of co-cultured MM-BMMSCs (n = 3) compared to mono-cultured MM-BMMSCs. Cyclin E1 was increased whereas cyclin D1 and p21 were reduced in co-cultured cells compared to mono-cultures. No change was seen for p16. **(D)** Cell interaction with KMS12-PE myeloma cells induced an increase of cells in G_1_/G_0_ phase and reduced the amount of cells in S phase in co-cultured and transwell cultured MM-BMMSCs (n = 8) compared to the same MM-BMMSCs cultured alone.

Co-cultures and transwell cultures of the KMS12-PE cell line with MM-BMMSCs were carried out to analyze whether MM cells can exert an influence on the senescence characteristics of MM-BMMSCs (Figure 3A). When co-cultured with myeloma cells MM-BMMSCs exhibited a reduced SA-βGalA compared to the same MM-BMMSCs cultured alone (median fluorescence intensity (MFI) of 118.1 vs. 178.4; *p <* 0.0001). A similar effect could be detected using transwell cultures to prevent cell-cell-contact between MM-BMMSCs and KMS12-PE cells (MFI of 173 vs. 267.3; *p <* 0.0313). No effect on the activity of SA-βGal was observed in HD-BMMSCs and the HS-5 cell line co-cultured with KMS12-PE myeloma cells. In addition, mRNA expression of co-cultured and transwell cultured MM-BMMSCs was measured (Figure 3B). No effect could be detected for cyclin D1 and p16, whereas cyclin E1 was 3.25-fold upregulated in co-cultured and 1.6-fold upregulated in transwell cultured MM-BMMSCs (*p <* 0.05). Surprisingly, cell interaction with MM cells also induced an upregulation of p21 mRNA in MM-BMMSCs. This effect was lower in transwell cultured MM-BMMSCs compared to co-cultured MM-BMMSCs (1.9-fold and 3-fold; *p <* 0.008). However, contrary results were detected at protein level showing a 1.7-fold reduction of p21 in co-cultured MM-BMMSCs (Figure 3C). In addition, cyclin D1 protein expression was 1.8-fold reduced upon co-cultivation with KMS12-PE myeloma cells whereas no change was seen on mRNA level (*p* = 0.0033). In contrast, the mRNA and protein analysis of cyclin E1 and p16 was concordant. Co-cultured MM-BMMSCs displayed also 2-fold upregulation of cyclin E1 on protein level against what the protein level of p16 was unchanged. In this context cell cycle distribution of co-cultured and transwell cultured MM-BMMSCs was analyzed (Figure 3D). Both cell culture systems led to a slight reduction of cells in S phase compared to MM-BMMSCs cultured alone (*p =* 0.0078). Furthermore, a light increase of cells in G_1_/G_0_ phase was detected for co-cultured MM-BMMSCs compared to MM-BMMSCs cultured alone (*p =* 0.0078). Transwell cultured MM-BMMSCs showed the same tendency but significant changes were not detectable.

### Cell interaction between KMS12-PE myeloma cells and MM-BMMSCs induces changes of microRNA expression in both cell types

**Figure 4:**
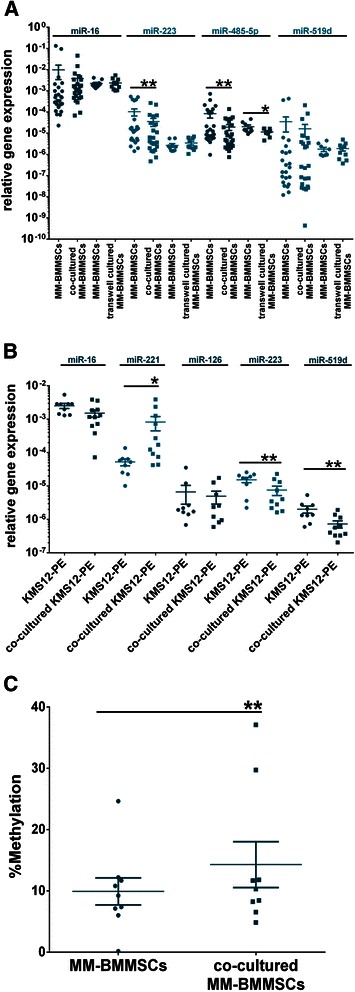
**KMS12-PE myeloma cells downregulate miR-223 and miR-485-5p in MM-BMMSCs.** Asterisks indicate indicate p-values with * <0.05, ** < 0.01, *** < 0.001 and **** < 0.0001. All data were analyzed using Wilcoxon signed-rank test. **(A)** Co-cultured MM-BMMSCs (n = 25) displayed reduced expression of miR-223 and miR-485-5p. Transwell-cultured (n = 10) MM-BMMSCs showed no changes in miR-223 expression but also decreased miR-485-5p levels. Intensity of changes in miR-485-5p decreased when cell-cell-contact was prevented by transwell cultivation. **(B)** Cell interaction with MM-BMMSCs induced changes in the microRNA expression of KMS12-PE myeloma cells (n = 10). MiR-221 was upregulated whereas miR-223 and miR-519d decreased in co-cultured KMS12-PE myeloma cells. **(C)** Cell interaction with KMS12-PE myeloma cells led to increased methylation of the IG-DMR of DLK1-DIO3 as measured by qMSP (n = 9).

To explore whether changes in senescence and cell cycle characteristics of MM-BMMSCs upon cultivation with KMS12-PE cells are associated with changes in the microRNA level of overexpressed microRNAs (miR-16, miR-223, miR-485-5p and miR-519d), the microRNA expression of co-cultured and transwell cultured MM-BMMSCs was measured using qPCR (Figure 4A). Significant changes were detected for miR-223 and miR-485-5p. MiR-223 was 3-fold downregulated in co-cultured MM-BMMSCs (*p <* 0.007), whereas no effect was detected in transwell cultured MM-BMMSCs. In contrast, downregulation of miR-485-5p was detected in both cell culture systems, whereby the effect reduced from 4-fold in co-cultured MM-BMMSCs to 2-fold in transwell cultured MM-BMMSCs (*p <* 0.03). Interestingly, cell-cell-interaction also altered microRNA expression of KMS12-PE myeloma cells. MiR-221 increased 13.78-fold whereas miR-223 and miR-519d were 2.72- to 2.85-fold reduced in co-cultured KMS12-PE myeloma cells (p < 0.02, Figure 4B). Expression of miR-485-5p was not detectable in KMS12-PE myeloma cells.

Furthermore, we analyzed whether changes in the methylation status of the DLK1-DIO3 regulatory region upon co-cultivation with KMS12-PE cells could be the reason for changes in miR-485-5p expression (Figure 4C). In co-cultured MM-BMMSCs a significant increase in the percentage IG-DMR methylation compared to MM-BMMSCs cultured alone was detected (14.31 ± 3.741% vs. 9.931 ± 2.21%; *p <* 0.004). In contrast, no CN variations of DLK1-DIO3 in co-cultured MM-BMMSCs compared to MM-BMMSCs cultured alone were detected (data not shown).

### MiR-485-5p modifies senescence and cell cycle characteristics of MM-BMMSCs

**Figure 5:**
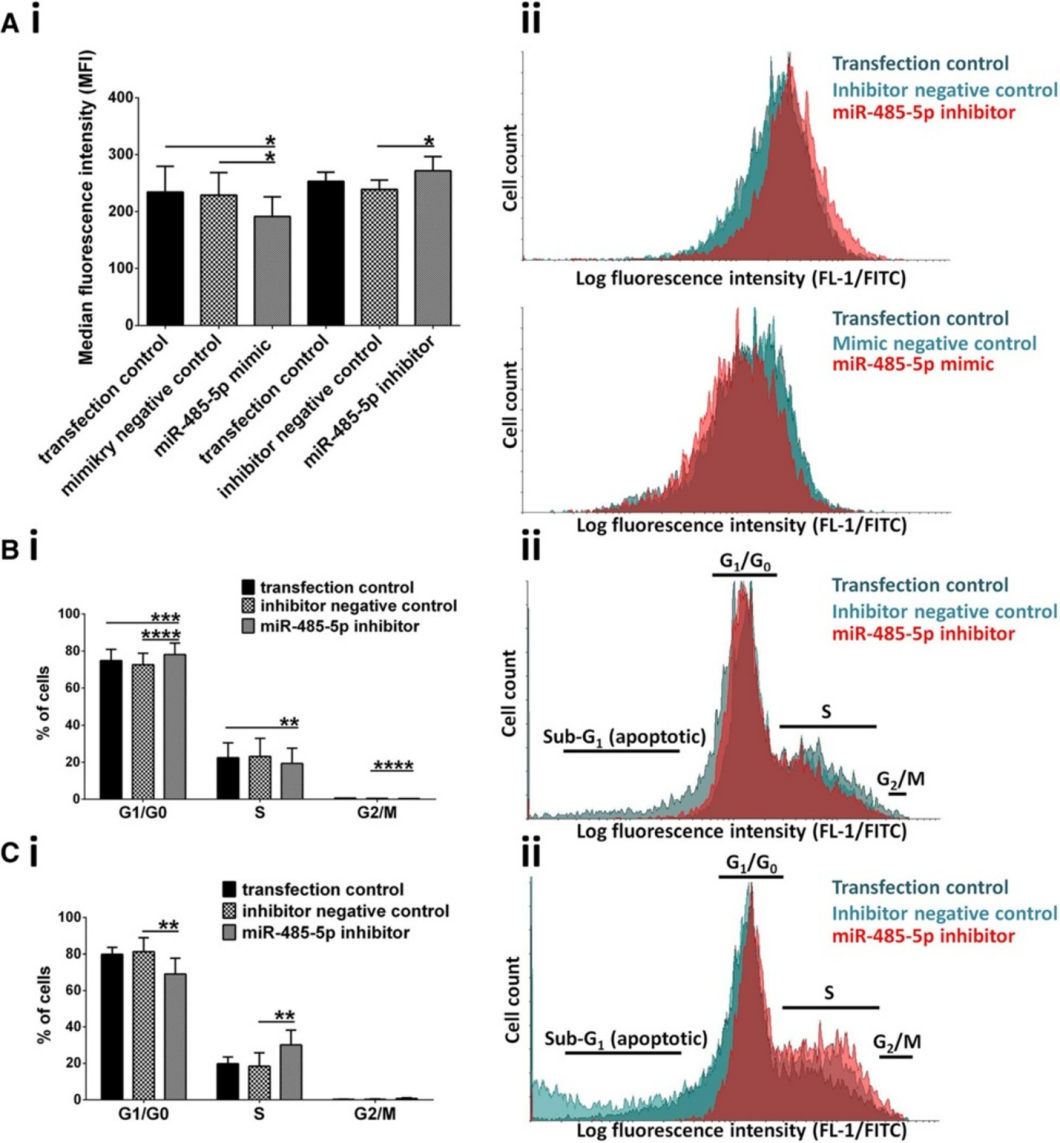
**miR-485-5p influences senescence and cell cycle characteristics of MM-BMMSCs.** Asterisks indicate indicate p-values with * <0.05, ** < 0.01, *** < 0.001 and **** < 0.0001. All data were analyzed using the paired t-test. **(Ai)** Transient overexpression of miR-485-5p led to reduced SA-βGalA whereas inhibition of miR-485-5p increased SA-βGalA. (ii) Representative histograms showing the effect of miR-485-5p inhibitor (n = 5) and miR-485-5p mimic (n = 5) on SA-βGalA in MM-BMMSCs compared to the transfection and negative control. **(Bi)** In three out of five transfected MM-BMMSCs the inhibition miR-485-5p induced the reduction of cells in S phase and accumulation of cells in G_1_/G_0_ phase of the cell cycle. (ii) Representative histogram showing one effect on the cell cycle of MM-BMMSCs induced by the inhibition of miR-485-5p. Reduced amount of cells in S phase is shown. **(Ci)** Two out of five transfected MM-BMMSCs displayed increased cells in S phase and reduced cells in G_1_/G_0_ phase upon transfection with the miR-485-5p inhibitor. (ii) Representative histogram showing the reverse effect on the cell cycle of MM-BMMSCs induced by the inhibition of miR-485-5p. Increased amount of cells in S phase is visible.

To explore whether downregulation of miR-485-5p is associated with the reduction of cells in S phase and SA-βGalA of MM-BMMSCs, transfections with miR-485-5p mimic or inhibitor were performed. Transient overexpression of miR-485-5p induced a reduction of SA-βGalA, whereas the inhibition of miR-485-5p led to an increase in the MFI compared to the negative and transfection controls (*p <* 0.05; Figure 5A). Overexpression of miR-485-5p had no effect on the cell cycle of MM-BMMSCs (data not shown), whereas inhibition of miR-485-5p induced different effects in MM-BMMSCs. Three MM-BMMSCs out of five transfected with the miR-485-5p inhibitor showed a decrease of cells in S phase and a significant increase of cells in G_1_/G_0_ phase compared to the controls (*p <* 0.003; Figure 5B). In contrast, two MM-BMMSCs displayed an inverse effect with an increase of cells in S phase and a decrease of cells in G_1_/G_0_ phase (*p <* 0.008; Figure 5C). These diverse results displayed no correlation with the ND-MM-BMMSCs or R-MM-BMMSCs characteristic.

## Discussion

MM-BMMSCs play a critical role in MM tumor growth and survival. Several studies suggest the existence of senescence-associated constitutive abnormalities in MM-BMMSCs, and that these lead to abnormal cell characteristics and increased tumor support [11-13,15,42,43]. In this study we investigated the correlation between senescence and microRNA expression in MM-BMMSCs and the influence of MM cells on these alterations.

In vitro cultured MM-BMMSCs showed increased senescence and accumulation of cells in S phase. These characteristics were associated with a reduced cyclin E1 and an increased cyclin D1 expression. R-MM-BMMSCs showed a higher SA-βGalA compared to ND-MM-BMMSCs. Therefore, it seems that relapse of MM could increase the premature senescence phenotype of MM-BMMSCs. In addition, the increased activity of SA-βGalA could be associated with the prolonged disease progression which is present in relapsed patients. As previously described we found overexpression of p21 but not p16 pointing to DNA damage mediated senescence [15]. Though senescence of MM-BMMSCs was already reported the reasons for this cellular state are not clear [12,14,15,43].

MicroRNAs are potential regulators of senescence but data relating to microRNA expression in MM-BMMSCs is limited [44]. Here, six microRNAs chosen on the basis of the microarray data from MM cells were analyzed [29]. Overexpression was found for miR-223, which was also overexpressed in MM cells. Furthermore miR-16, miR-485-5p and miR-519d were overexpressed in MM-BMMSCs whereas these microRNAs were downregulated in MM cells. Previous studies have identified miR-16 as a tumor suppressor in leukemic cells and its overexpression in MM-BMMSCs could induce a similar effect [45]. In contrast, no specific functions of miR-223, miR-485-5p and miR-519d in MM are known. MiR-485-5p and miR-519d, which are expressed on the clusters DLK1-DIO3 and C19MC, were reported to play a role in senescence generation [21,32,46,47]. The regulatory regions of both clusters were hypomethylated together with CN accumulation of the genomic regions in MM-BMMSCs. These two alterations could be responsible for the overexpression of miR-485-5p and miR-519d. The aberration of genomic CN also points to genomic alterations in MM-BMMSCs possibly explaining the p21-associated senescence state. In addition, Abdeolmohsen et al. reported that miR-519 promotes DNA damage and elevates the level of p21 [32].

Next, we analyzed whether cell interaction with MM cells influences these alterations. Surprisingly, cell interaction with KMS12-PE cells led to reduced SA-βGalA and cells in S phase of the cell cycle. This effect was also seen when cell-cell-contact was prevented by transwells. Thus, the increase of cells in G_1_/G_0_ phase points to a pro-proliferative influence of MM cells on MM-BMMSCs. This shift in the cell cycle characteristics could also be the reason for the decreased cyclin D1 expression and the increased expression of cyclin E1 in co-cultured MM-BMMSCs. Noll et al. reported that MM cells alter the microenvironment of MM by supporting the proliferation of mesenchymal stem cells through soluble factors as IL-6 [48]. Therefore oncogenic signaling by MM cells by different cytokines could induce the reversal of senescence in MM-BMMSCs [49]. In addition, the reduction of SA-βGalA was accompanied by a reduction of p21 at the protein level. In contrast, p21 mRNA expression was increased. The mRNA amount is not a direct reflection of protein expression since post-transcriptional processes are significant to the final synthesis of proteins. Contrary expression levels of mRNA and proteins could be of interest for the understanding of the interaction between myeloma cells and BMMSCs in MM pathogenesis. Here, it could be induced by a feedback mechanism activated through the depletion of p21 at the protein level. In addition, the reversal mechanisms of senescence are still not clear [17,18]. Even though senescence in MM-BMMSCs seems to be generated by increased p21 expression it is possible that reversal of this cellular state is mediated by a distinct signaling pathway [31,45].

Cell interaction with MM cells reduced levels of miR-223 and miR-485-5p in MM-BMMSCs. Given that only miR-485-5p was also reduced when cell-cell-contact between MM-BMMSCs and MM cells was prevented, this microRNA was a potential candidate for modulation of senescence in MM-BMMSCs. In addition, cell interaction seems to increase methylation of the IG-DMR of DLK1-DIO3 possibly leading to reduced expression of the cluster-associated microRNAs [50]. Interaction of KMS12-PE myeloma cells with MM-BMMSCs also induced microRNA changes in the MM cell fraction. Cell-cell-interaction strongly increased the level of miR-221 in KMS12-PE myeloma cells. This microRNA acts as an oncogene by downregulation of different cell cycle inhibitors leading to an increased tumor cell proliferation [51]. In addition, miR-223 and miR-519d were downregulated in co-cultured KMS12-PE myeloma cells. As previously mentioned these microRNAs could have anti-proliferative effects on cells [21,32,46,47]. Therefore, interaction with MM-BMMSCs seems to shift microRNA levels of KMS12-PE myeloma cells towards a more pro-proliferative gene expression pattern.

Finally, MM-BMMSCs were transfected with miR-485-5p mimic or inhibitor. The effect on SA-βGalA was contrary to the one seen by cell interaction with MM cells because increased miR-485-5p levels decreased the SA-βGalA and vice versa. This indicates that miR-485-5p alone is not responsible for reversal of senescence in MM-BMMSCs but additional genetic events are needed. This could be based on the fact that increased methylation of the IG-DMR of DLK1-DIO3, which possibly leads to reduced transcription of this genomic region, controls all associated microRNAs. Therefore, the mechanism which induces the observed effect in MM-BMMSCs upon cell interaction with MM cells could be rather complex. Interestingly, inhibition of miR-485-5p also induced different cell cycle effects in MM-BMMSCs. Three of the transfected MM-BMMSCs displayed an effect which was similar to the one induced by cell interaction with MM cells, whereas the other two showed a contrary cell cycle change. This again indicates that miR-485-5p acts in dependence of additional molecular genetic events or cellular properties [47].

Although manipulation of miR-485-5p alone could not mimic the effect on SA-βGalA and the cell cycle of MM-BMMSCs upon cell interaction with MM cells, the results strongly indicate that miR-485-5p and the associated cluster DLK1-DIO3 participate in the regulation of senescence and cell cycle characteristics in MM-BMMSCs. Therefore, further investigations of this topic are needed. Furthermore, the premature senescence phenotype of MM-BMMSCs seems to be an attendant phenomenon rather than a specific tumor supportive stromal cell characteristic. The reduction of the senescence phenotype by MM cells indicates that interaction of MM cells and MM-BMMSCs does not rely on this cell characteristic. It is likely that MM cells benefit from a more vital cellular state of MM-BMMSCs. Therefore, the premature senescence phenotype of MM-BMMSCs could be a side effect possibly induced through increased activation of stromal cells by MM cells. However, further analysis is needed to determine whether the senescence-associated constitutive changes of MM-BMMSCs could support relapse of MM disease.

## Conclusions

In summary we found that senescence in MM-BMMSCs is associated with the overexpression of potential senescence-mediating microRNAs and copy number variations of DLK1-DIO3 and C19MC. This is the first report indicating that the senescence phenotype of MM-BMMSCs is partially reversed by cell interaction with MM cells and it is therefore not clear whether this phenotype could in vivo be important for the pathogenesis of active MM disease or is more important when cell interaction of MM-BMMSCs with MM cells is inhibited. More investigations regarding the role of senescence of MM-BMMSCs in MM could lead to new insights for the improvement of therapy efficiency and follow-up treatment of MM patients. The genomic region of DLK1-DIO3 includes potential senescence-modulating microRNAs and further analysis of the role of DLK1-DIO3 and the associated microRNAs could reveal new insights into the genetic events that senescence relies on.

## Additional files

Additional file 1: Table S1. Patients and Donor characteristics.
Additional file 2: Figure S1. Purity of isolated BMMSCs. BMMSCs were double stained with CD34-PE/CD90-FITC or CD45-PE/CD105-FITC. Purity of BMMSCs ranged from 94% to 99.5% in passage 1 of cell cultures.
Additional file 3: Table S2. Primers used in this study. **Table S3.** Cycling conditions for qPCR. **Table S4.** qBiomarker Assays used for CN variation analysis.

## Abbreviations

MM: Multiple myeloma
MM-BMMSCs: Multiple myeloma bone marrow stromal cells
ND-MM-BMMSCs: Newly diagnosed MM-BMMSCs
R-MM-BMMSCs: Relapsed MM-BMMSCs
SA-βGalA: Senescence-associated β-galactosidase activity
