# Supplementary material for: Multiple myeloma cells alter the senescence phenotype of bone marrow mesenchymal stromal cells under participation of the DLK1-DIO3 genomic region
Source: BMC Cancer. 2015 Feb 18;15:68. doi: 10.1186/s12885-015-1078-3 (PMC4336751; doi:10.1186/s12885-015-1078-3)
Supplement: Additional file 1: Table S1. — Patients and Donor characteristics. [file 12885_2015_1078_MOESM1_ESM.pdf]

Additional File 1

Table S1: Patients and Donor characteristics

Patients/Donors	Total Patients (n=89)	Newly diagnosed MM (ND-MM-BMMSCs; n=54)	Relapsed MM (R-MM-BMMSCs; n=35)	Donors (n=12)
Median Age	64.5 (33-85)	66 (33-85)	62 (44-84)	65 (43-75)
Sex (n; count)				
Male	56 (62.9%)	35 (64.8%)	21 (60.0%)	8 (66.7%)
Female	33 (37.1%)	19 (35.2%)	14 (40.0%)	4 (33.3%)
Paraprotein isotype				
IgG		32 (59.3%)	18 (51.4%)	
IgA		8 (14.8%)	3 (8.6%)	
IgD		0 (0%)	1 (2.8%)	
light chain		13 (24.1%)	13 (37.2%)	
asecretory		1 (1.8%)	0 (0%)	
Durie/Salmon state				-
I		12 (22.2%)	2 (5.8%)	
II		7 (13.0%)	8 (22.8%)	
III		35 (64.8%)	25 (71.4%)	
Bone marrow infiltration [%]		45 (10-90)	80 (10-100)	-
