# Supplementary material for: Multiple myeloma cells alter the senescence phenotype of bone marrow mesenchymal stromal cells under participation of the DLK1-DIO3 genomic region
Source: BMC Cancer. 2015 Feb 18;15:68. doi: 10.1186/s12885-015-1078-3 (PMC4336751; doi:10.1186/s12885-015-1078-3)
Supplement: Additional file 2: Figure S1. — Purity of isolated BMMSCs. BMMSCs were double stained with CD34-PE/CD90-FITC or CD45-PE/CD105-FITC. Purity of BMMSCs ranged from 94% to 99.5% in passage 1 of cell cultures. [file 12885_2015_1078_MOESM2_ESM.pdf]

Additional File 2: Purity of isolated BMMSCs

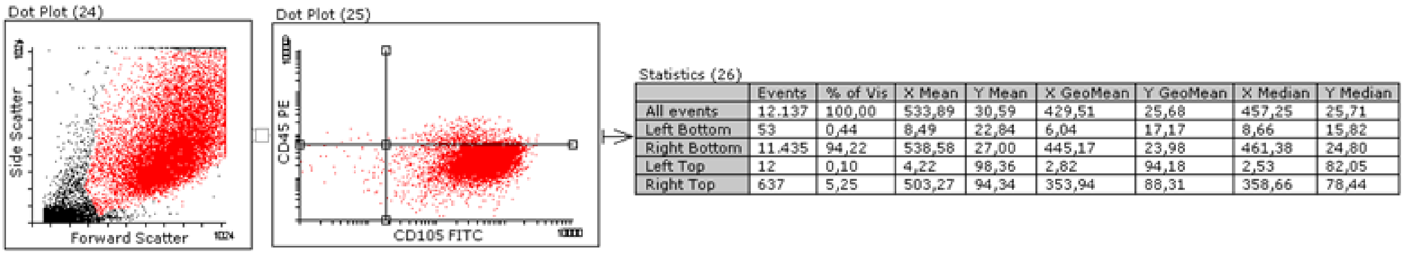

Statistics (26)

	Events	% of Vis	X Mean	Y Mean	X GeoMean	Y GeoMean	X Median	Y Median
All events	12.137	100,00	533,89	30,59	429,51	25,68	457,25	25,71
Left Bottom	53	0,44	8,49	22,84	6,04	17,17	8,66	15,82
Right Bottom	11.435	94,22	538,58	27,00	445,17	23,98	461,38	24,80
Left Top	12	0,10	4,22	98,36	2,82	94,18	2,53	82,05
Right Top	637	5,25	503,27	94,34	353,94	88,31	358,66	78,44

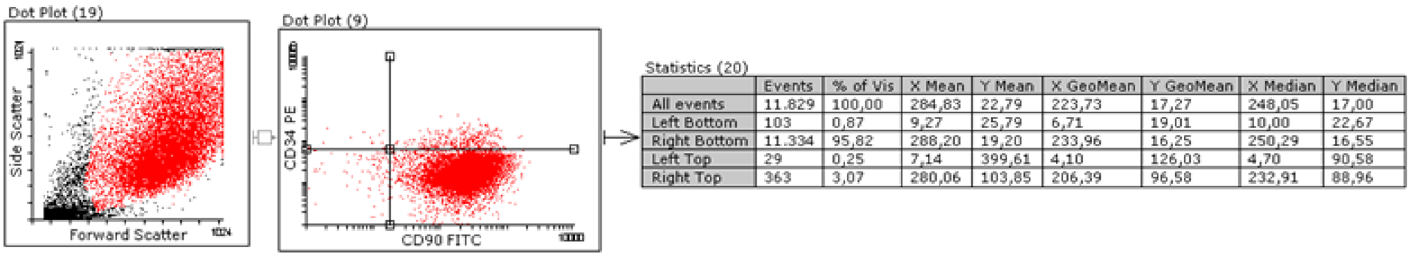

Statistics (20)

	Events	% of Vis	X Mean	Y Mean	X GeoMean	Y GeoMean	X Median	Y Median
All events	11.829	100,00	284,83	22,79	223,73	17,27	248,05	17,00
Left Bottom	103	0,87	9,27	25,79	6,71	19,01	10,00	22,67
Right Bottom	11.334	95,82	288,20	19,20	233,96	16,25	250,29	16,55
Left Top	29	0,25	7,14	399,61	4,10	126,03	4,70	90,58
Right Top	363	3,07	280,06	103,85	206,39	96,58	232,91	88,96

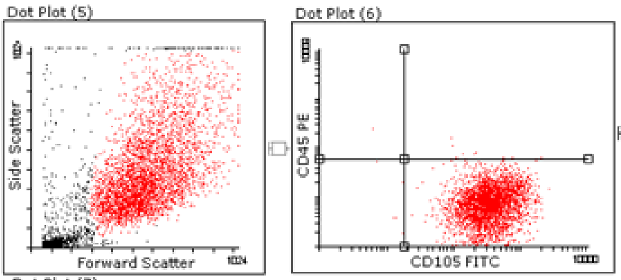

Statistics (27)

	Events	% of Vis	X Mean	Y Mean	X GeoMean	Y GeoMean	X Median	Y Median
All events	3.738	100,00	417,05	9,38	339,81	6,86	342,89	7,43
Left Bottom	6	0,16	11,64	15,70	8,56	8,42	12,86	3,89
Right Bottom	3.722	99,57	417,70	9,13	342,43	6,81	342,89	7,37
Left Top	1	0,03	6,21	235,01	6,21	235,01	0,00	0,00
Right Top	9	0,24	463,61	83,13	255,59	79,39	138,24	64,36

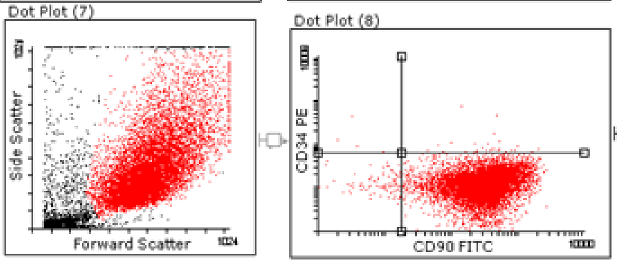

Statistics (28)

	Events	% of Vis	X Mean	Y Mean	X GeoMean	Y GeoMean	X Median	Y Median
All events	10.529	100,00	390,06	14,18	289,93	11,50	316,23	11,86
Left Bottom	106	1,01	9,80	14,72	7,53	12,71	10,55	11,44
Right Bottom	10.379	98,58	394,00	13,65	301,88	11,39	319,08	11,86
Left Top	8	0,08	7,08	282,35	4,09	140,27	3,31	81,31
Right Top	36	0,34	459,82	105,54	307,89	83,52	345,99	69,78

Figure S1: Purity of isolated BMMSCs. BMMSCs were double stained with CD34-PE/CD90-FITC or CD45-PE/CD105-FITC. Purity of BMMSCs ranged from 94% to 99.5% in passage 1 of cell cultures.
