# Supplementary material for: Multiple myeloma cells alter the senescence phenotype of bone marrow mesenchymal stromal cells under participation of the DLK1-DIO3 genomic region
Source: BMC Cancer. 2015 Feb 18;15:68. doi: 10.1186/s12885-015-1078-3 (PMC4336751; doi:10.1186/s12885-015-1078-3)
Supplement: Additional file 3: Table S2. — Primers used in this study. Table S3. Cycling conditions for qPCR. Table S4. qBiomarker Assays used for CN variation analysis. [file 12885_2015_1078_MOESM3_ESM.pdf]

Additional File 3: Supplemental Methods

Table S2: Primers used in this study

Name	Sequence	Application	Fragment size	Accession number*
Poly(T)-VN	GCGAGCACAGAATTAATACGACTCACTATAGG(T)$_{12}$VN*	cDNA synthesis	-	-
Cyclin E1	5´-CCTGGATGTTGACTGCCTTG<br>5´- ATGTCGCACCACTGATACCC	qPCR	114	NM_001238
Cyclin D1	5´- GATCAAGTGTGACCCGGACT<br>5´-GGGTCCATGTTCTGCTGG	qPCR	96	NM_053056
p16	5´- CACCAGCGTGTCCAGGAAG<br>5´- GCCGATCCAGGTCATGATG	qPCR	128	NM_00077/<br>NM_001195132
p21	5´- CACTGTCTTGTACCCTTGTGC<br>5´- GGTAGAAATCTGTCATGCTGG	qPCR	114	NM_001220778/<br>NM_001220777/<br>NM_078467/<br>NM_000389/<br>NM_001291549
GAPDH	5´- GAAATCCCATCACCATCTTCC<br>5´- CAAATGAGCCCCAGCCTTC	qPCR	123	NM_002046/<br>NM_001289745/<br>NM_001289746/<br>NM_001256799
hsa-miR-16	5´- TAGCAGCACGTAAATATTGGCGAA<br>5´- GCGAGCACAGAATTAATACGAC	qPCR	58	MI0000070
hsa-miR-221	5´- GCTACATTGTCTGCTGGGTTTC<br>5´- GCGAGCACAGAATTAATACGAC	qPCR	56	MI0000298
hsa-miR-126	5´- TCGTACCGTGAGTAATAATGCG<br>5´- GCGAGCACAGAATTAATACGAC	qPCR	56	MI0000471

hsa-miR-223	5´- TGTCAGTTTGTCAAATACCCCAAA<br>5´- GCGAGCACAGAATTAATACGAC	qPCR	58	MI0000300
hsa-miR-485-5p	5´- GGCTGGCCGTGATGAATTC<br>5´- GCGAGCACAGAATTAATACGAC	qPCR	53	MIMAT0002175
hsa-miR-519d	5´- CAAAGTGCCTCCCTTTAGAGTG<br>5´- GCGAGCACAGAATTAATACGAC	qPCR	56	MI0003162
5.8 S rRNA	5´- CTACGCCTGTCTGAGCGTCGCTT<br>5´- GCGAGCACAGAATTAATACGAC	qPCR	57	NR_003285
DLK1-DIO3<br>(methylated)	5´- GTTAGTAATCGGGTTTGTCGGC<br>5´- AATCATAACTCCGAACACCCGCG	qMSP	160	-
DLK1-DIO3<br>(unmethylated)	5´- GAGGATGGTTAGTTATTGGGGT<br>5´- CCACCATAACCAACACCCTATAATCACA	qMSP	120	-
C19MC<br>(methylated)	5´- TGACGTCGTTACGTTTTGGA<br>5´- TAAACCGAAAAACGCGCG	qMSP	106	-
C19MC<br>(unmethylated)	5´- TGATGTTGTTATGTTTTGGATTGAG<br>5´- AAACACACACCAACACCTCATAACA	qMSP	97	-

*** according to UCSC, NCBI and miRBase**

Table S3: Cycling conditions for qPCR

Step	Temperature [°C]	Time [s]	Cycles
Denaturation	95	600	
Denaturation	95	15	x 45
Annealing	59-60	60	
Melt	60-98	ΔT +1°C/s	

Table S4: qBiomarker Assays used for CN variation analysis

Cluster	Assay	Genomic Region	Gene
DLK1-DIO3	VPH114-0506637A	chr14:101327201-101327400	MEG3
	VPH114-0507099A	chr14:101419601-101419800	LOC100507242
	VPH114-0507682A	chr14:101536201-101536400	LOC100507257
C19MC	VPH119-0270702A	chr19:54140201-54140400	DPRX
	VPH119-0271054A	chr19:54210601-54210800	MIR520C
	VPH119-0307173A	chr19:54296838-54297302	NLRP12
Normalisation	VPH000-0000000A		
